# Antimicrobial activity of ceftaroline against methicillin-resistant *Staphylococcus aureus* (MRSA) isolates collected in 2013–2014 at the Geneva University Hospitals

**DOI:** 10.1007/s10096-016-2807-5

**Published:** 2016-10-15

**Authors:** D. O. Andrey, P. François, C. Manzano, E. J. Bonetti, S. Harbarth, J. Schrenzel, W. L. Kelley, A. Renzoni

**Affiliations:** 1 Service of Infectious Diseases, Department of Medical Specialties, Geneva University Hospitals and Medical School, Geneva, Switzerland; 2 Genomic Research Laboratory, Service of Infectious Diseases, Department of Medical Specialties, Geneva University Hospitals and Medical School, Geneva, Switzerland; 3 Infection Control Program, Geneva University Hospitals and Medical School, Geneva, Switzerland; 4 Bacteriology Laboratory, Department of Laboratories and Genetic Medicine, Geneva University Hospitals, Geneva, Switzerland; 5 Department of Microbiology and Molecular Medicine, Faculty of Medicine, Geneva University, Geneva, Switzerland; 6 Service of Infectious Diseases, Geneva University Hospital and Medical School, 4 Rue Gabrielle Perret Gentil, Geneva, Switzerland

## Abstract

Ceftaroline is a broad-spectrum antibiotic with activity against methicillin-resistant *Staphylococcus aureus* (MRSA) strains. Ceftaroline susceptibility of an MRSA set archived between 1994 and 2003 in the Geneva University Hospitals detected a high percentage (66 %) of ceftaroline resistance in clonotypes ST228 and ST247 and correlated with mutations in PBP2a. The ceftaroline mechanism of action is based on the inhibition of PBP2a; thus, the identification of PBP2a mutations of recently circulating clonotypes in our institution was investigated. We analyzed ceftaroline susceptibility in MRSA isolates (2013 and 2014) and established that resistant strains correlated with PBP2a mutations and specific clonotypes. Ninety-six MRSA strains were analyzed from independent patients and were isolated from blood cultures (23 %), deep infections (38.5 %), and superficial (skin or wound) infections (38.5 %). This sample showed a ceftaroline minimum inhibitory concentration (MIC) range between 0.25 and 2 μg/ml and disk diameters ranging from 10 to 30 mm, with a majority of strains showing diameters ≥20 mm. Based on the European Committee on Antimicrobial Susceptibility Testing (EUCAST) breakpoints, 76 % (73/96) of isolates showed susceptibility to ceftaroline. Nevertheless, we still observed 24 % (23/96) of resistant isolates (MIC = 2 μg/ml). All resistant isolates were assigned to clonotype ST228 and carried the N146K mutation in PBP2a. Only two ST228 isolates showed ceftaroline susceptibility. The decreasing percentage of ceftaroline-resistant isolates in our hospital can be explained by the decline of ST228 clonotype circulating in our hospital since 2008. We present evidence that ceftaroline is active against recent MRSA strains from our hospital; however, the presence of PBP2a variants in particular clonotypes may affect ceftaroline efficacy.

## Introduction

Infections caused by methicillin-resistant *Staphylococcus aureus* (MRSA) are a major worldwide health problem. MRSA, initially identified as a nosocomial pathogen, is now responsible for both hospital- and community-acquired infections [1]. MRSA treatment options include glycopeptides and combination regimens [2], as well as new agents like daptomycin and last-generation cephalosporins (ceftobiprole and ceftaroline). Despite the availability of several antimicrobials, resistance to a particular antibiotic has repeatedly brought complications for the successful treatment of MRSA infections.

β-Lactam antibiotics were first developed to inhibit penicillin-binding proteins (PBPs) that catalyze cell wall biosynthesis. However, soon after their introduction, the efficacy of β-lactams was altered by *S. aureus* strains producing a β-lactamase enzyme or by horizontal acquisition of *mecA*, encoding the penicillin-binding protein PBP2a. PBP2a is not inhibited by β-lactams and can consequently catalyze DD-transpeptidation and cooperate with the PBP2 transglycosylase in peptidoglycan biosynthesis [3, 4].

Ceftaroline is a novel β-lactam broad-spectrum cephalosporin, capable of inhibiting PBP2a. Its anti-MRSA activity shows an MIC_90_ of around 1–2 μg/ml and has been approved for the treatment of complicated skin and soft tissue infections and community-acquired pneumonia [5–7]. Several studies have shown decreased susceptibility of MRSA to ceftaroline (EUCAST MIC values of > 1 μg/ml or CLSI MIC ≥ 4 μg/ml) in sporadic cases and limited to specific sequence types (STs) [8–12]. Recently, high-level ceftaroline resistance (MIC > 32 μg/ml) was observed during sustained MRSA bacteremia treated with ceftaroline [13]. Low-level resistance to ceftaroline is associated with mutations in PBP2a found in both the allosteric domain (N146K, E150K, N204K, E239K, G246E) or the transpeptidase domain (H351N, Y446N, E447) [3, 8, 13–17].

While high-level ceftaroline resistance can be explained by mutations in the transpeptidase domain that alter the active site geometry, low-level resistance associated with mutations within the allosteric site was explained by a novel allosteric modulation of the active site [15, 18–20]. Ceftaroline binds to the PBP2a allosteric site at therapeutic concentrations and triggers the opening and acylation of the active site by a second ceftaroline molecule [7, 20]. Mutations within the PBP2a allosteric domain alter the triggering mechanism and possibly affect protein–protein interactions needed for peptidoglycan biosynthesis [7, 14, 19, 21–23]. Additional factors contributing to ceftaroline resistance remain to be analyzed [24]. Surprisingly, reduced susceptibility has been described in clinical *S. aureus* isolates predating the commercial introduction of ceftaroline [8, 25] and in countries where ceftaroline is not commercially available [17].

Our previous study showed that decreased ceftaroline susceptibility was linked to circulating clonotypes ST228 and ST247 carrying mutations in the allosteric domain of PBP2a [8]. Since there has been a major change in MRSA epidemiology at our institution during the last 5 years [26], the analysis of ceftaroline susceptibility and mutations in PBP2a of contemporary circulating MRSA isolates in our institution was undertaken. MRSA isolates from 2013 to 2014 causing either soft tissue infections or bloodstream infections were examined.

## Materials and methods

### Bacterial strains and susceptibility testing

A collection of 96 independent MRSA strains archived from 2013 to 2014 and representing bloodstream, deep tissue, and superficial infection samples were tested. The methicillin-susceptible *S. aureus* (MSSA) and ceftaroline-susceptible strain ATCC29213, MIC 0.25 μg/ml, was used as the EUCAST standard quality control strain. Standardized procedures were as previously described [8] and determined according to EUCAST recommendations.

### *mecA* gene sequencing

Genomic DNA (gDNA) was prepared as previously described [27] and *mecA* was amplified by polymerase chain reaction (PCR) (3 Kb) using specific primers: seq_meclocus-F 5′ TAAGGGAGAAGTAACAGCAC 3′ and seq_meclocus-R 5′ ATCGCCCAAAGCTTCTTTAG 3′. PCR fragments were sequenced using appropriate primers within *mecA*. Sequence alignments were performed using Clustal Omega and *mecA* reference sequences from N315 (SA0038) and COL (SACOL0033).

### MLVA genotyping

Each tested MRSA was analyzed by a rapid genotyping assay (MLVA) [28]. The genotype of each strain was deduced by comparison with profiles obtained with well-characterized standard isolates.

## Results

### Determination of ceftaroline susceptibility in the MRSA strain collection (2013–2014) of Geneva University Hospitals

A previous study from our laboratory found a high rate of ceftaroline non-susceptibility in a collection of MRSA strains recovered from bloodstream infections during the period 1994–2003 [8]. To determine if ceftaroline resistance was still prevalent in our institution, we examined the ceftaroline susceptibility of 96 MRSA isolates archived in 2013–2014, using both disk diffusion and microdilution MIC methods. The specimen sources included 37 deep wound infections (38.5 %), 37 superficial (skin or wound) infections (38.5 %), and 22 bloodstream infection (23 %) isolates. Using ceftaroline 5 μg disks, our strain collection showed mean diameters ranging from 10 to 30 mm (Table 1), with a majority (79 %) of isolates (76/96) showing diameters ≥20 mm, but 21 % of isolates (20/96) showing a diameter <20 mm in at least three independent measurements (EUCAST breakpoints S ≥ 20 and R < 20 mm).

**Table 1:** Ceftaroline minimum inhibitory concentration (MIC) values and sequence types (STs) of 96 methicillin-resistant *Staphylococcus aureus* (MRSA) strains (Geneva University Hospitals 2013–2014) tested by microdilution and disk diffusion assays

Strain number	Archived date	^b^Ceftaroline MIC (μg/ml)		^c^Ceftaroline 5 μg disk diameter (mm)	^d^Susceptible phenotype	ST
		24 h	48 h			
1	2013	2	2	20	R	228
2	2013	1	1	21	S	105
3	2013	0.5	0.5	24	S	8
4	2013	1	1	22	S	105
5	2013	2	2	18	R	228
6	2013	2	4	17	R	228
7	2013	1	1	21	S	105
8	2013	0.25	0.5	25	S	8
9	2013	0.5	0.5	22	S	105
10	2013	1	1	21	S	105
11	2013	0.5	0.5	22	S	8
12	2013	1	1	22	S	228
13	2013	0.5	0.5	22	S	30
14	2013	1	1	21	S	5
15	2013	2	2	16	R	228
16	2013	0.25	0.5	25	S	80
17	2013	0.5	0.5	24	S	8
18	2013	0.5	0.5	25	S	8
19	2013	0.5	0.5	23	S	8
20	2013	0.5	0.5	24	S	8
21	2013	0.5	0.5	22	S	80
22	2013	0.5	0.5	24	S	8
23	2013	2	4	17	R	228
24	2013	2	4	17	R	228
25	2013	0.25	0.5	23	S	NT
26	2013	0.5	1	22	S	8
27	2013	2	4	18	R	228
28	2013	2	2	17	R	228
29	2013	1	1	21	S	105
30	2013	0.5	0.5	23	S	8
31	2013	0.5	1	26	S	105
32	2013	2	4	18	R	228
33	2013	0.5	0.5	25	S	125
34	2013	0.5	0.5	23	S	105
35	2013	0.5	1	21	S	80
36	2013	0.5	0.5	22	S	108
37	2013	0.5	1	24	S	8
38	2013	0.5	1	23	S	8
39	2013	2	2	19	R	228
40	2013	0.5	0.5	22	S	5
41	2013	0.5	0.5	23	S	8
42	2013	2	4	17	R	228
43	2013	2	4	17	R	228
44	2013	2	2	18	R	228
45	2013	0.5	0.5	22	S	30
46	2013	2	4	18	R	228
47	2013	2	4	19	R	228
48	2013	0.5	0.5	24	S	NT
49	2013	0.5	0.5	23	S	105
50	2013	1	1	22	S	105
51	2013	2	4	16	R	228
52	2013	0.5	0.5	23	S	30
53	2013	0.5	0.5	22	S	30
54	2013	0.5	0.5	23	S	8
55	2013	0.5	0.5	24	S	30
56	2013	0.5	0.5	23	S	8
57	2013	0.5	0.5	25	S	8
58	2013	2	4	17	R	228
59	2013	0.5	0.5	24	S	8
60	2013	2	4	10	R	228
61	2013	0.5	0.5	22	S	105
62	2013	0.5	0.5	24	S	8
63	2013	0.5	0.5	28	S	NT
64	2013	0.5	0.5	25	S	NT
65	2013	0.5	0.5	24	S	8
66	2013	2	4	14	R	228
67	2013	0.5	0.5	23	S	80
68	2013	0.5	0.5	23	S	8
69	2013	0.5	0.5	22	S	30
70	2013	0.5	1	23	S	5
71	2013	0.25	0.5	25	S	5
72	2013	0.5	1	23	S	8
73	2014	0.25	0.5	26	S	30
74	2014	2	4	20	R	228
75	2014	1	2	22	S	228
76	2014	1	1	22	S	80
77	2014	2	4	18	R	228
78	2014	0.5	0.5	23	S	8
79	2014	0.25	0.5	30	S	8
80	2014	2	2	19	R	228
81	2014	2	4	20	R	228
82	2014	0.5	0.5	23	S	30
83	2014	0.5	0.5	23	S	105
84	2014	0.5	0.5	25	S	125
85	2014	0.5	0.5	24	S	8
86	2014	1	1	23	S	105
87	2014	0.5	0.5	27	S	105
88	2013	0.5	0.5	24	S	105
89	2013	1	1	23	S	105
90	2013	1	2	21	S	125
91	2013	0.25	0.5	26	S	30
92	2013	1	1	23	S	5
93	2013	0.5	0.5	25	S	5
94	2013	1	1	25	S	105
95	2014	0.25	0.5	25	S	1
96	2013	0.5	0.5	24	S	1
^a^ATCC29213		0.25	0.5	29	S	

If the diameter is between 19 and 21 mm, an MIC determination will confirm susceptibility

*NT* non-typable strain

^a^MIC values for ATCC29213 are within the EUCAST-approved ranges for ceftaroline

^b^EUCAST microdilution ceftaroline susceptibility breakpoint S ≤ 1; R > 1

^c^EUCAST ceftaroline disk susceptibility breakpoints S ≥ 20 mm; R < 20 mm

^d^Susceptibility phenotype based on EUCAST recommendation

MIC determinations are required to confirm the susceptibility of strains showing disk diameters between 19 and 21 mm, according to the EUCAST guidelines. All strains were tested by the microdilution assay by three independent measurements (Table 1). For all strains tested, the disk diffusion results were in agreement with the MIC microdilution assays. The isolates with diameters 19 or 20 mm were all confirmed to be resistant by MIC microdilution, while those showing diameters of 21 mm were all confirmed as susceptible.

Overall, this strain set showed a ceftaroline MIC range between 0.25 and 2 μg/ml and 0.25 and 4 μg/ml at 24 and 48 h of incubation, respectively (Table 1). The frequency of MIC distribution for ceftaroline was 8.3, 52.1, 15.6, and 24 % for isolates showing an MIC of 0.25, 0.5, 1, and 2 μg/ml, respectively. A large proportion (73/96; 76 %) of isolates showed susceptibility to ceftaroline (EUCAST microdilution breakpoints S ≤ 1 and R > 1 μg/ml), with the majority (50/96) showing an MIC value of 0.5 μg/ml. Nevertheless, we still observed an elevated proportion (23/96; 24 %) of isolates showing an MIC of 2 μg/ml (at 24 h), thus considered resistant (Table 1).

### PBP2a allosteric site mutations correlate with reduced ceftaroline susceptibility

The previously published ceftaroline-resistant strains from our hospital (the 1994–2003 collection) principally belong to clonotypes ST228 and ST247 [8]. The most plausible hypothesis to explain the presence of resistant strains in our 2013–2014 collection is that they represent bacterial clones belonging to the ST228 or ST247 typing family and carrying PBP2a mutations [8, 25]. Accordingly, MRSA isolates were subjected to MLST determination and PBP2a (*mecA*) sequence analysis. Isolates were first subjected to multiple-locus variable-number tandem repeat analysis (MLVA), a rapid genotyping assay allowing assessment of genomic content and assignment of MLST type [28, 29]. The STs of all strains are presented in Table 1.

PBP2a (*mecA*) sequence analysis was next performed (Table 2). PBP2a sequences from our strain collection were compared to the previously published Greek strain sequences and amino acid changes were annotated [16] (Table 2). Interestingly, and consistent with our hypothesis, all resistant strains (MIC = 2 μg/ml) were unambiguously assigned to ST228 and carried the PBP2a N146K mutation. In contrast, the majority of susceptible strains were all assigned to other STs (ST1, ST5, ST8, ST30, ST80, ST93, ST105, and ST125). Of note, two isolates from the 25 ST228 isolates show ceftaroline susceptibility, but also carry the N146K mutation, suggesting that this mutation alone might not be sufficient to generate ceftaroline resistance in certain circumstances. We found no other PBP2a mutation in our ceftaroline-resistant subset. In the ceftaroline-susceptible non-ST228 isolates, the only PBP2a mutations identified were G246E, found in four isolates (3 out of 10 *mecA* sequenced isolates). No G246E mutation was found in ceftaroline-resistant isolates. This corroborates other studies where no influence on the ceftaroline susceptibility of this mutation could be associated with ceftaroline susceptibility profiles [15, 17].

**Table 2:** PBP2a mutation profiles and ST of selected strains

Strain collection	Archived date	Strain number	Strain name	ST	PBP2a mutations							
					MIC	N146K	E150K	E239K	N240K	G246E	H351N	E447K
^a^Greece			4981	ST5	1							
^a^Greece			4977	ST239	2	K	K		K	K		
^a^Greece	2008		13101	ST239	4	K	K		K	K	N	
HUG	2013	1	1437	ST228	2	K						
HUG	2013	5	1441	ST228	2	K						
HUG	2013	6	1442	ST228	2	K						
HUG	2013	7	1443	ST105	1					E		
HUG	2013	12	1448	ST228	1	K						
HUG	2013	15	1478	ST228	2	K						
HUG	2013	21	1484	ST80	0.5					E		
HUG	2013	23	1486	ST228	2	K						
HUG	2013	24	1487	ST228	2	K						
HUG	2013	27	1490	ST228	2	K						
HUG	2013	28	1491	ST228	2	K						
HUG	2013	32	1495	ST228	2	K						
HUG	2013	34	1497	ST105	0.5					E		
HUG	2013	37	1500	ST8	0.5							
HUG	2013	39	1502	ST228	2	K						
HUG	2013	42	1505	ST228	2	K						
HUG	2013	43	1506	ST228	2	K						
HUG	2013	46	1509	ST228	2	K						
HUG	2013	47	1510	ST228	2	K						
HUG	2013	51	1514	ST228	2	K						
HUG	2013	52	1515	ST30	0.5							
HUG	2013	58	1521	ST228	2	K						
HUG	2013	59	1522	ST8	0.5							
HUG	2013	60	1523	ST228	2	K						
HUG	2013	66	1529	ST228	2	K						
HUG	2013	69	1532	ST30	0.5							
HUG	2014	74	1537	ST228	2	K						
HUG	2014	75	1538	ST228	1	K						
HUG	2014	77	1540	ST228	2	K						
HUG	2014	80	1543	ST228	2	K						
HUG	2014	81	1544	ST228	2	K						
HUG	2014	85	1548	ST93	0.5							
HUG	2013	90	1553	ST125	1							
HUG	2014	95	1558	ST1	0.25							
HUG			COL	ST250	0.25					E		
HUG			N315	ST5	1							

^a^Mendes et al. [16]

### Analysis by sample type

We found ceftaroline-resistant isolates irrespective of the anatomical specimen source. The resistance analysis by sample type reveals that 31.8 % (7/22) of resistant bacteria were isolated from bloodstream infections, 29.7 % (11/37) from deep infections, and 13.5 % (5/37) from superficial soft tissue/wound infections. Comparing isolates from superficial infections with a resistance rate of 13.5 % (5/37) to more invasive isolates with a resistance rate of 30.5 % (18/59), it might be inferred that more severe infections are correlated to higher resistance levels. This result is linked to the higher prevalence of the ST228 clonotype in deep wounds and bloodstream infection than in superficial wound specimens. Thus, interestingly, the ST228 hospital-acquired MRSA strain is more frequent in bloodstream infections and deep wound infections than in community-acquired infections, such as skin and superficial wound infection.

## Discussion

Ceftaroline-resistant strains (MIC of 2 μg/ml) were previously reported and identified in a few clonotypes, including ST228, ST247, and ST239 [8, 15, 21, 25, 30]. In 2015, we reported a high percentage (66 %) of ceftaroline resistance in an HUG bacterial collection first assembled to monitor the emergence of intermediate glycopeptide resistance [8, 31]. The detection of ceftaroline resistance among ST228 and ST247 clonotypes in this archived set prompted us to examine contemporary MRSA strains from our institution, not biased by prior screening for intermediate glycopeptide resistance. To what extent recent ST228 strains display reduced susceptibility to ceftaroline is an important consideration. Our current study was further motivated by the fact that ST228 has been endemic since 1998 in our institution, but epidemiological changes have been observed over the last 5–8 years, with gradual decline of ST228 and replacement by other clonotypes [14, 32] and, furthermore, by the potential use in clinical practice of this drug licensed in Switzerland in 2013, but which has not been used in our hospital to date.

The majority of 2013–2014 MRSA tested in the present study were found to be susceptible to ceftaroline, showing a modal MIC of 0.5 μg/ml; however, we observed 24 % resistant isolates (MIC = 2 μg/ml). The percentage of resistance we report in both studies is subject to cautious interpretation, since the current strain set may not be representative of an unbiased sampling since only certain infection types were analyzed. A second explanation for the lower percentage of resistant strains in the 2013–2014 compared to the 1994–2003 strain set can be the fact that resistance appears to be strongly linked with ST228, a clonotype gradually declining in recent years in our hospital [14, 32].

Although the majority of tested 2013–2014 HUG strains were found to be susceptible to ceftaroline, it is noteworthy that all resistant isolates were ST228. Ceftaroline-resistant HUG ST228 strains are strongly correlated with the N146K mutation present in the PBP2a allosteric domain. Together with E150K and E239K, the N146K mutation is one of the most prevalent mutations in isolates exhibiting a ceftaroline MIC of 2 μg/ml (including Italy, Hungary, Russia, Spain, and Turkey) [15, 22]. This mutation was previously detected in the ST228 bacterial strains collected in the interval 1998–2003 in our hospital and through 2003–2008 in the University Hospital of Lausanne [8]. It is unknown how the South German clone present in our hospital acquired PBP2a mutation. Whether some MRSA clonotypes, such as ST228, are more amenable to genetic variation is unknown. Since different missense mutations in PBP2a are found in several ST228 strains, it is probable that independent events occurred to produce this observed PBP2a allotype variation [8, 15]. ST228, even if diminishing in prevalence over the last decade in Switzerland, was still responsible for a large part of the non-community-acquired MRSA infections in the 2013–2014 period, which represents a significant concern.

Mutations in the allosteric domain of PBP2a are thought to confer low-level ceftaroline resistance by gating the active site channel [19]. In some cases, genotypic analysis revealed mutation within *mecA*, but the strain, nevertheless, displayed susceptibility to ceftaroline [8]. An important consideration is that proper transcriptional expression of *mecA*, together with PBP2a’s posttranslational export, maturation, and positioning, are crucial steps that govern PBP2a function. Whereas the transcriptional regulation of *mecA* is clearly multifactorial [4], only recently have reports highlighted the importance of additional factors such as PrsA that affect PBP2a maturation [33, 34]. Taken in this light, the presence of certain mutations within the PBP2a allosteric domain may not evoke resistance because of expression constraints. Alternatively, some strains showing a ceftaroline MIC of 2 μg/ml show wild-type PBP2a sequence, raising the possibility that additional factors can influence ceftaroline susceptibility. Notably, mutations in *clpX*, *stp1*, and *prsA*, as well as the *pbp4* promoter, appear to be associated with ceftaroline resistance [24, 34]. Further genetic studies must identify genes implicated in ceftaroline resistance and define how particularly allosteric domain mutations differentially impact the expression of ceftaroline resistance.
